# The effect of extended participation windows on attendance at cervical cancer screening

**DOI:** 10.1016/j.pmedr.2023.102166

**Published:** 2023-02-27

**Authors:** Kelly M. Castañeda, Grigory A. Sidorenkov, Jolien de Waard, Marcel J.W. Greuter, Bert van der Vegt, Inge M.C.M. de Kok, Albert G. Siebers, Karin M. Vermeulen, G. Bea A. Wisman, Ed Schuuring, Geertruida H. de Bock

**Affiliations:** a Department of Epidemiology, University of Groningen, University Medical Center Groningen, 9700 RB Groningen, the Netherlands; b Department of Gynaecologic Oncology, Cancer Research Center Groningen, University of Groningen, University Medical Centre Groningen, 9700 RB Groningen, the Netherlands; c Department of Radiology, University of Groningen, University Medical Center Groningen, 9700 RB Groningen, the Netherlands; d Department of Pathology & Medical Biology, University of Groningen, University Medical Centre Groningen, 9700 RB Groningen, the Netherlands; e Department of Public Health, Erasmus MC, University Medical Center Rotterdam, 3000 CA Rotterdam, the Netherlands; f PALGA, the Nationwide Network and Registry of Histo- and Cytopathology in the Netherlands, 3991 SZ Houten, the Netherlands

**Keywords:** CCS, cervical cancer screening, hrHPV, high-risk human papillomavirus, PALGA, Dutch Nationwide Pathology Databank, Uterine cervical neoplasms, Early detection of cancer, Patient compliance

## Abstract

Research has long since confirmed the benefits of regular cervical cancer screening (CCS) worldwide. However, some developed countries have low participation rates despite well-organized screening programs. Given that studies in Europe typically define participation in 12-month windows from an invitation, we evaluated both whether extending this defined time window could reveal the true participation rate and how sociodemographic determinants affect participation delays. This involved linking data from the Lifelines population-based cohort with CCS-related data from the Dutch Nationwide Pathology Databank and including data for 69 185 women eligible for screening in the Dutch CCS program between 2014 and 2018. We then estimated and compared the participation rates for 15- and 36-month time windows and categorized women by the primary screening window into timely participation (within 15 months) and delayed participation (within 15–36 months) groups, before performing multivariable logistic regression to evaluate the association between delayed participation and the sociodemographic determinants. Participation rates for the 15- and 36-month windows were 71.1% and 77.0%, respectively, with participation considered timely in 49 224 cases and delayed in 4047 cases. Delayed participation was associated with age 30–35 years (odds ratio [OR]: 2.88, 95 %CI: 2.67–3.11), higher education (OR: 1.50, 95 %CI: 1.35–1.67), the high-risk human papillomavirus test-based program (OR: 1.67, 95 %CI: 1.56–1.79), and pregnancy (OR: 4.61, 95 %CI: 3.88–5.48). These findings show that a 36-month window for monitoring attendance at CCS better reflects the actual participation rate by accommodating possible delayed uptake among younger, pregnant, and highly educated women.

## Highlights

• Participation rates in cervical cancer screening are usually estimated using time windows of 15 months or shorter.
• The participation rate increases significantly when using a 36-month time window.
• Younger age, pregnancy, and higher education are associated with delayed participation.

## 1 Introduction

Implementing cervical cancer screening (CCS) programs in Europe has contributed to reducing the mortality associated with the disease (Jansen et al., 2020, Sung et al., 2021). Nevertheless, many countries with established programs, such as France, Denmark, and the Netherlands, have national coverage rates that fall short of the 70% threshold recommended by the World Health Organization to ensure an efficient CCS program (Maver and Poljak, 2020, Coverage of national cervical cancer screening program, 2022, World Health Organization, 2021). This is problematic when we consider that invasive disease in such countries presents mostly in women who do not take part in screening (Arbyn et al., 2018, Bos et al., 2006), with low participation observed particularly in younger women, immigrants, and those of low socioeconomic status (Aitken et al., 2021, Audiger et al., 2021, Harder et al., 2018).

Implementing robust surveillance and monitoring systems is key to identifying gaps in reducing cervical cancer incidence and mortality (Bruni et al., 2022). The coverage rate, defined as the number of screened women in the total eligible population in a given time interval, is a core indicator that reflects the capacity to provide testing for primary screening at a country level (Bruni et al., 2022). However, the definition of the time interval is arbitrary and can change by country. Most studies in Europe have defined participation using time windows of 12 months after an invitation (Aitken et al., 2021, Audiger et al., 2021, Hermens et al., 2000), whereas the Dutch CCS estimates the participation rate using a 15-month window from the start of the invitation year (National Institute for Public Health and the Environment, 2017). However, evidence from a recent report has indicated that a short time window for monitoring the Dutch CCS might not be enough to capture all participation, especially since implementing the new high-risk human papillomavirus (hrHPV)-based screening program that introduced many organizational changes (Aitken, 2021). Extending the participation window could give a better indication of the true participation rate by reflecting possible delays.

The present study targets these issues with two primary aims. First, we assessed whether extending the time window for participation from 15 months to 36 months could increase the participation rate, based on the trends in 2014–2018. Second, we evaluated the association between sociodemographic determinants and participation delays.

## 2 Material and methods

### 2.1 Study design

This study used a cross-sectional design nested in a population-based cohort. We linked data from the Lifelines cohort (Scholtens et al., 2015) and the Dutch Nationwide Pathology Databank (PALGA) (Casparie et al., 2007) between 2014 and 2018. Lifelines is a multi-disciplinary prospective population-based cohort study examining in a unique three-generation design the health and health-related behaviours of 167 729 persons living in the North of the Netherlands. It employs a broad range of investigative procedures in assessing the biomedical, socio-demographic, behavioural, physical and psychological factors which contribute to the health and disease of the general population, with a special focus on multi-morbidity and complex genetics (Scholtens et al., 2015, Stolk et al., 2008). PALGA provides centralized data from all pathology laboratories in the Netherlands (Casparie et al., 2007). We used data from PALGA to estimate the participation rate in the CCS based on the primary screening date recorded as part of the organized screening program within either 15 or 36 months of the start of the invitation year, and to compare women who had a record within 15 months and 15–36 months after the start of the invitation year, defining these as timely and delayed participants, respectively.

### 2.2 Study population

The Lifelines cohort included 167 729 people between December 2006 and December 2013. It set out to follow them for at least 30 years, with follow-up questionnaires every 1.5 years and physical examinations every 5 years (Scholtens et al., 2015) Currently, Lifelines has completed the baseline questionnaire and a physical assessment (2007–2013), three follow-up questionnaires (2011–2014, 2012–2015, 2016–2019), and a second physical assessment (2014–2017), with the third physical assessment ongoing (2019–2023) (Lifelines Wiki, 2020).

The CCS-related data from PALGA between January 2000 and December 2020 were linked to all women in the Lifelines cohort based on a combination of the family name, date of birth, sex, and zip code. For this project, we accessed the following data from the PALGA database: cytology, hrHPV positivity, histology records, nature of testing (primary or secondary screening), and the reason for testing (organized screening or another indication). Before 2017, screening organizations invited women aged 30–60 years for primary cytology testing every 5 years (Aitken et al., 2019). Since then, they have invited women for primary hrHPV testing at ages 30, 35, 40, 50, and 60 years, only inviting those aged 45, 55, and 65 years if they are hrHPV positive or missed their last screening (National Institute for Public Health and the Environment, 2021). Because HPV statuses were unknown at the start of the hrHPV-based program, all women aged 30–60 years were invited to the first round (2017–2021) (National Institute for Public Health and the Environment, 2021). Due to these factors and a considerable decline in the participation rate since introducing the hrHPV-based program (Aitken et al., 2021), we selected a screening round of 5 years to reflect both versions of the program when evaluating delays in participation. Therefore, women who turned 30, 35, 40, 45, 50, 55, and 60 years old in each year between 2014 and 2018 were considered eligible for CCS and included in the analyses. We excluded women with any hysterectomy (based on self-report in the Lifelines questionnaire before 2000 and PALGA records from 2000 onwards) or who had died before the invitation year (based on Lifelines questionnaires).

**Ethical approval**

Informed consent was obtained from all participants. The Lifelines study complies with the principles of the Declaration of Helsinki and it received approval from the Medical Ethics Committee of the University Medical Center Groningen, the Netherlands (no**.** 2007/152)**.**

### 2.3 Determinants

The assessed sociodemographic determinants comprised the invitation year, age that year, country of birth, ethnicity, educational level, income level, marital status, and pregnancy during the invitation year. All determinants (except pregnancy) were derived from the self-reported baseline questionnaires for the Lifelines cohort. The invitation year was categorized as 2014–2016 and 2017–2018 to compare the cytology-based and hrHPV-based programs, respectively. Age in the year of invitation was estimated by the year of birth. Educational level was measured according to the highest academic level achieved and was categorized according to the standard categorization of educational level in the Netherlands, as follows: low (no education, primary education, lower or preparatory vocational education, lower general secondary education), middle (intermediate vocational education or apprenticeship, higher general senior secondary education or pre-university secondary education), and high (higher vocational education, university) (van Zon et al., 2018). Income was recorded as low, medium, and high when the net income per month was less than €1500, between €1500 and €2500, and higher than €2500, respectively, or as unknown (“I don’t know,” “I don’t want to say,” or did not respond or missing response) (Faruque et al., 2021). Due to the substantial number of missing entries for country of birth and ethnicity, we combined the data as follows: those reported as white-European ethnicity with missing values for the country of birth were considered born in the Netherlands (n = 425); and those reported as white Mediterranean, Arabic, black, and Asian were considered born in other countries (n = 6). Only 0.2% (158/69 185) of women had missing values for both ethnicity and country of birth. If the invitation year included one of the following two distinct periods based on a biological child’s birth date, we assumed the woman was pregnant that year: (1) from 9 months before the birth date until 3 months after the birth date, including the dates women reported being pregnant in the follow-up questionnaires of the Lifelines study; and (2) from the questionnaire date to 3 months after.

### 2.4 Statistical analysis

To assess whether extending the time for the definition of CCS participation significantly increased the participation rate, we estimated the proportion of eligible women who participated per year with the respective 95% confidence intervals. Rates were calculated as the number of women with a primary screening record in PALGA within either 15 months or 36 months of the start of the invitation year, divided by the total number of women eligible in the invitation year. Paired-sample *t-*tests were used to estimate possible statistically significant differences between the participation rates at 15 months and 36 months. To evaluate the association between sociodemographic determinants and participation timeliness, we first presented the sociodemographic determinants by the timely and delayed participant groups from among all eligible women. Secondly we included all women who had a primary screening record in PALGA within 36 months after the start of the invitation year in the univariable and multivariable logistic regression analyses, using the previously mentioned determinants as covariables and participation as the outcome (delayed compared with timely). Before carrying out the multivariable analysis, we performed a Spearman correlation test to evaluate whether including education and income could generate collinearity in the multivariable model. All analyses were conducted using IBM SPSS Version 25.0 (IBM Corp., Armonk, NY, USA).

## 3 Results

In total, 69 185 of 89 176 women from the Lifelines cohort were eligible for primary CCS between 2014 and 2018. The average participation rate was higher using a 36-month window (77.0%; 95 %CI: 76.7–77.3) compared with a 15-month window (71.1%; 95 %CI: 70.8–71.5) (P < 0.001; Table 1). When using the 15-month window, the participation rate decreased over time from 73.1% in 2014 to 68.4% in 2018, but it increased again to 69.9% in 2019. However, when using a 36-month window, the participation rate changed less markedly (79.0% in 2014; 77.9% in 2015; 76.0% in 2016; stable at approximately 76% in subsequent years; Table 1).

**Table 1:** CCS participation in the Lifelines cohort from 2014 to 2018 by time window.

Invitation year	Women eligible for screening ^a^ n	Participation rate		Difference in participation rate (P value)^d^
		15-month window ^b^ % [95 %CI] (n)	36-month window ^c^ % [95 %CI] (n)	
2014	13 802	73.1 [72.3–73.8] (10 088)	79.0 [78.3–79.7] (10 901)	5.9 (<0.001)
2015	13 850	73.0 [72.2–73.7] (10 104)	77.9 [77.2–78.6] (10 795)	4.9 (<0.001)
2016	14 144	71.4 [70.7–72.2] (10 100)	76.0 [75.3–76.7] (10 746)	4.6 (<0.001)
2017	13 813	68.4 [67.6–69.2] (9 445)	75.9 [75.2–76.6] (10 481)	7.5 (<0.001)
2018	13 576	69.9 [69.1–70.7] (9 487)	76.2 [75.5–76.9] (10 348)	6.3 (<0.001)
**Total**	**69 185**	**71.1 [70.8–71.5] (49 224)**	**77.0 [76.7–77.3] (53 271)**	**5.9 (<0.001)**

^a^Women eligible for screening: Number of women in Lifelines aged 30/35/40/45/50/55/60 after excluding women who had a hysterectomy and died before the Invitation year.

^b^Number of women with at least one primary screening record in PALGA from January first of the year of eligibility till April first of the following year (15 months) divided the total number of women eligible in the year of eligibility.

^c^Number of women with at least one primary screening record in PALGA from January first of the year of eligibility till 36 months after divided the total number of women eligible in the year of eligibility.

^d^Paired-sample T-Test.

Table 2 presents the sociodemographic determinants by participation window. In this cohort, 5.9% of women had delayed participation in the CCS, but with 14% of women aged 30–35 years having delays compared with only 4.8% of those aged 40–50 years and 2.4% of those aged 55–60 years. Around 7% of women invited to CCS in 2017–2018 had delayed participation compared with about 5% invited in 2014–2016. By the country of birth, delays occurred in 5.9% and 4.7% of the women born in the Netherlands and in other countries, respectively (11.4% who did not report country of birth and/or ethnicity had delays). About 8% of highly educated women took part with delays, while fewer than 6% of those with middle and low education levels had delays. More single women had delayed participation compared with women in the other marital status categories. The distribution of delayed participation was similar by income category. Finally, 32.4% of women who were pregnant during the invitation year had delayed participation, compared with only 5.5% of those who were not pregnant.

**Table 2:** Sociodemographic determinants by timely and delayed participation in the CCS for the 2014–2018 period.

Determinants		Total women eligible	Timely participants n (%)	Delayed participants n (%)
Total		69 185	49 224 (71.1)	4047 (5.9)
*Age at the Invitation year*				
30–35		13 087	8134 (62.2)	1829 (14.0)
40–45–50		36 395	26 433 (72.6)	1735 (4.8)
55–60		19 703	14 657 (74.4)	483 (2.5)
*Invitation year*				
2014–2016 (cytology)		41 796	30 292 (72.5)	2150 (5.1)
2017–2018 (HPV)		27 389	18 932 (69.1)	1897 (6.9)
*Country of birth*				
The Netherlands		66 412	47 586 (71.7)	3907 (5.9)
Other country		2615	1553 (59.4)	122 (4.7)
Missing		158	85 (53.8)	18 (11.4)
*Educational level*				
Low		16 292	11 703 (71.8)	549 (3.4)
Middle		29 469	21 239 (72.1)	1651 (5.6)
High		22 245	15 466 (69.5)	1774 (8.0)
Missing		1179	816 (69.2)	73 (6.2)
*Income*				
Low		9754	6478 (66.4)	630 (6.5)
Medium		16 120	11 288 (70.0)	983 (6.1)
High		29 922	21 861 (73.1)	1839 (6.1)
Unknown		13 389	9597 (71.7)	595 (4.4)
*Marital status*				
Married/relationship		39 130	28 321 (72.4)	2137 (5.5)
Single		3 935	2 337 (59.4)	306 (7.8)
Divorced		352	250 (71.0)	<10 (<2.0)
Widow		1895	1328 (70.1)	58 (3.1)
*Pregnancy during invitation year*				
No		68 386	48 860 (71.4)	3 788 (5.5)
Yes		799	364 (45.6)	259 (32.4)

We categorized women by the primary screening window into timely participation (within 15 months) and delayed participation (within 15–36 months) groups.

Missing are not reported for marital status to protect the confidentiality of the participants.

Table 3 shows the associations between participant characteristics and participation window for only those women with a screening record within 36 months after the start of invitation year (n = 53 271). Univariable analysis revealed that all putative covariables were significant. Spearman’s correlation coefficient between education level and income was 0.059, indicating a low probability of collinearity. Therefore, all determinants could be included as variables in the multivariable model. Multivariable logistic regression showed associations between delayed participation and age, invitation year, education level, and pregnancy. Women aged 30–35 years were almost three times as likely to have delayed participation as women aged 40 years or older. Those invited in 2017–2018 were also more likely to be included after a delay than those invited in 2014–2016. Having a middle or higher educational level was also associated with delayed participation compared a low educational level. Finally, pregnancy during the invitation year had the largest effect on delayed participation, with these women being 4.6 times more likely to have delays than women who were not pregnant.

**Table 3:** Univariable and multivariable analyses of the determinants of delayed versus timely participation in the CCS for the 2014–2018 period.

Determinants	Univariable model		Multivariable model ^a^	
	OR (95 %CI)	P value	OR (95 %CI) ^a^	P value
*Age*		<0.001		<0.001
30–35	3.43 (3.20–3.68)		2.88 (2.67–3.11)	
40–45–50	Ref.		Ref.	
55–60	0.50 (0.45–0.56)		0.50 (0.45–0.56)	
*Invitation year*		<0.001		<0.001
2014–2016 (Cyt)	Ref.		Ref.	
2017–2018 (HPV)	1.41 (1.32–1.51)		1.67 (1.56–1.79)	
*Country of birth*		0.001		0.014
The Netherlands	Ref.		Ref.	
Other country	0.96 (0.79–1.15)		1.03 (0.85–1.25)	
Missing	2.583 (1.55–4.30)		2.41 (1.33–4.37)	
*Educational level*		<0.001		<0.001
Low	Ref.		Ref.	
Middle	1.66 (1.50–1.83)		1.20 (1.08–1.33)	
High	2.45 (2.22–2.70)		1.50 (1.35–1.67)	
Missing	1.91 (1.48–2.46)		1.30 (0.97–1.74)	
*Income*		<0.001		0.119
Low	Ref.		Ref.	
Medium	0.90 (0.81–0.99)		1.02 (0.92–1.14)	
High	0.87 (0.79–0.95)		1.00 (0.90–1.11)	
Unknown	0.64 (0.57–0.72)		0.90 (0.79–1.02)	
*Marital status*		<0.001		0.258
Married/relationship	Ref.		Ref.	
Single	1.74 (1.53–1.97)		1.08 (0.94–1.25)	
Divorced	0.37 (0.18–0.79)		0.67 (0.32–1.43)	
Widow	0.58 (0.44–0.76)		0.80 (0.61–1.06)	
Missing	1.20 (1.12–1.29)		1.02 (0.95–1.09)	
*Pregnancy during Invitation year*		<0.001		<0.001
No	Ref.		Ref.	
Yes	9.18 (7.80–10.80)		4.61 (3.88–5.48)	

Data are shown as odds ratios (ORs) and 95% confidence intervals (95% CIs). P-values are based on Wald test.

^a.^ Model. Age + Invitation year + country of birth + Educational level + Income + Marital status + Pregnancy during invitation year.

## 4 Discussion

In this representative cohort from the north of the Netherlands, we show that changing the definition of participation by extending the time window for considering CCS participation increased the estimated participation rate from 71.1% to 77.0% between 2014 and 2018. Moreover, we identified age 30–35 years, middle or high educational level, pregnancy during the invitation year, and invitation year as determinants of delayed participation.

This study shows a decrease in participation from 2014 to 2015 to 2016–2018 for both the 15-month and 36-month time windows. This is consistent with the known decrease in participation since introducing the hrHPV-based program (Aitken et al., 2021). However, the participation rate in this study was much higher among inhabitants from the north of the Netherlands than in earlier reports. At a national level, the participation rate decreased by 7.8% from 64.8% to 57% over the 2014–2017 period (Centrum et al., 2016, Integraal Kankercentrum Nederland, 2020), whereas in the present study, it decreased by 4.7% from 73.1% to 68.4% for the same period and 15-month window. Extending the participation window to 36-months led to a reduction of 3.1% (from 79% in 2014 to 75.9% in 2017) in the participation rate for 2014–2017 in the north of the Netherlands. Such a minor change might not have important implications for the CCS in this region because the total participation rate, including delays, exceeded 75% for the 2014–2018 period. The population distribution in the Netherlands might explain these differences, with the north having a lower number of immigrants than other regions (Centraal Bureau voor de Statistiek, 2022). In 2018, for example, only 5% of all immigrants to the Netherlands lived in the north, compared with 21% in the east, 53% in the west, and 21% in the south (Centraal Bureau voor de Statistiek, 2022). Studies tend to report lower participation rates in immigrants due to language barriers and cultural differences (Bongaerts et al., 2020, Chorley et al., 2017, Idehen et al., 2020, Marques et al., 2020). Thus, the Lifelines cohort probably lacks these barriers, and if so, will present a higher participation rate. Another explanation could be that women from Lifelines are more willing to take part in a population-based study, which may make them more inclined to take part in the CCS. Further research is needed to evaluate the impact of delayed participation on national data.

For this study, an extension of a 15 to 36-month window was used to assess whether the time definition could significative increase the participation rate. However, as the program is every five years, a 60-months window would cover the actual total participation rate. Therefore, we used a 36-month window for two main reasons. 1) Since we only had data until December 2020, the maximum time to follow women invited in 2018 is 36 months. 2) When we compared the participation rate using a 36 and 60-month window, the difference was only 0,01 from 2014 to 2016.

Although we are aware of no research that has discussed the determinants of delayed participation, research has mentioned the possible impact of the definition of participation. Indeed, a short time window for monitoring the Dutch CCS was considered potentially insufficient, especially since introducing the new hrHPV-based screening program that required many organizational changes (Aitken, 2021). Our project shows that extending the time window when defining participation may produce a more accurate estimate of the actual participation rate, especially since 2017.

Regarding the determinants for delayed participation, since women with delays are, by time definition, wrongly counted as non-participants, in this study, we included determinants for non-participation as well as pregnancy. Our analysis showed that younger women were more likely to delay participation in the CCS compared with older women. Even though age and pregnancy during the invitation was independently associated with delayed participation, a reason for delayed participation at a younger age could be that the average age when women have their first child has been increasing in the Netherland since 2015, with a current average of 30 years (Centraal Bureau voor de Statistiek, 2013). In fact, pregnancy had the largest effect on delayed participation, which is a direct consequence of the way the cervical cancer screening is organized. During pregnancy there is no screening for cervical cancer in the Netherlands and the invitation for pregnant women will be sent six months after the delivery (National Institute for Public Health and the Environment, 2021). As a result, women are still considered non-participants in a short time window when they may not have even completed the time to be screened. In addition, the number of highly educated women in the Netherlands has increased in recent year, such that around 50% of women aged 25–35 years were deemed highly educated in 2018 (Centraal Bureau voor de Statistiek, 2019). These sociodemographic changes in education, pregnancy, and age might affect the time of participation because young, highly educated, or pregnant women might not have the time to take part in the CCS within the current 15-month window, but eventually, they do. Although country of birth does not appear to have a major role in participation delay, women who did not report their country of birth or ethnicity were at higher odds of delay. Nevertheless, only 0.2% (158/69 185) of women had missing data on this variable, making this association trivial.

This study benefited from being conducted in a large population-based cohort with extensive follow-up data from the north of the Netherlands, ensuring representativeness for the general population only in this region (Klijs et al., 2015). The CCS data were also retrieved from PALGA, an automated pathology databank with national coverage (Casparie et al., 2007), so this study could rely on a complete history of cervical diagnostics, including cytology, hrHPV test results, and histological diagnoses from colposcopies and treatment procedures. This allowed us to define the population at risk more precisely by identifying and excluding women with hysterectomies. PALGA also provides accurate and standardized pathology data for the annual monitoring of the Dutch CCS program (Casparie et al., 2007). Even though it may be widely accepted that pregnancy affects participation in CCS, this study confirms the actual role of pregnancy based on data collected in Lifelines.

This study has some important limitations. First, lack of knowledge of the exact day on which an invitation was sent meant that we used a period from January 1st of the invitation year based on the usual procedure for monitoring CCS in the Netherlands (National Institute for Public Health and the Environment, 2017). This decision might have led to information bias because women could have had more or less time to take part in the screening. However, we expect a random distribution of this information bias, with it not significantly affecting the results because screening organizations sent invitations either randomly during the year (before 2017) or a few days after a birthday (since 2017). Considering the 36-month time window, all women had a minimum time of 2 years to participate, regardless of the exact invitation date. Second, the use of self-sampling might have played a role in participation delay because patients were only offered a self-sampling test if they did not take part by 4 months after the first invitation. Nonetheless, only 2.5% (1729/69 185) of the women in the current study attended through self-sampling; given that 68% of these were in the timely participant group, this may not have affected the overall results. Third, we used baseline measurements collected in 2006–2013 from the Lifelines cohort to determine educational level, income, and marital status (Scholtens et al., 2015), yet this study evaluated participation for the 2014–2018 period. Because the mean age of participants in Lifelines was 41 years at baseline (Scholtens et al., 2015), we expect neither substantial changes in these variables after inclusion nor a significant impact on the study findings. Fourth, when evaluating pregnancy during the invitation year, we only considered pregnancies that resulted in live births (according to the birth date of the children) or those reported during follow-up in the Lifelines cohort (self-reported current pregnancy when completing the questionnaire). By not considering any miscarriages or abortions that occurred between assessments, we may have underestimated the effect size of pregnancy on participation delays in the CCS. Fifth, although we combined the country of birth with ethnicity to reduce missing data, we do not expect this to affect the result drastically because Lifelines is a very homogenous white European cohort (Scholtens et al., 2015, Klijs et al., 2015). Finally, the linkage between the PALGA and Lifelines databases could have introduced bias by linking based on the family name, date of birth, sex, and zip codes initials especially for women with the same family names. Nevertheless, in a large cohort that also includes the zip code initials, these errors are unlikely.

Our study shows that a 15-month window to monitor the screening program might not cover the actual participation rate in the CCS because delays in participation are likely to occur in younger, pregnant, highly educated women. Since non-participation is an outcome of high importance for the effectiveness of an organized screening program, the time definition should be carefully sett-up to identify precisely the gaps to reduce cervical cancer mortality. Future studies should consider extending the time window to get a precise estimation of the participation rate, and, therefore, a better definition of non-participants.

## 5 Conclusion

Using a time window of 36 months instead of 15 months to monitor participation in the Dutch CCS program resulted in a higher participation rate. Determinants for delayed participation in the CCS were younger age, pregnancy, middle/higher education, and invitation year. Even though participation in the north of the Netherlands was higher than at the national level, extending the time window in the Netherlands could more accurately reflect the true participation rate.

## CRediT authorship contribution statement

**Kelly M. Castañeda:** Conceptualization, Data curation, Methodology, Formal analysis, Writing – original draft. **Grigory A. Sidorenkov:** Conceptualization, Methodology, Writing – review & editing. **Jolien de Waard:** Conceptualization, Writing – review & editing. **Marcel J.W. Greuter:** Writing – review & editing. **Bert van der Vegt:** Writing – review & editing. **Inge M.C.M. de Kok:** Writing – review & editing. **Albert G. Siebers:** Methodology, Writing – review & editing. **Karin M. Vermeulen:** Conceptualization, Writing – review & editing. **G. Bea A. Wisman:** Supervision, Conceptualization, Writing – review & editing. **Ed Schuuring:** Supervision, Conceptualization, Writing – review & editing. **Geertruida H. de Bock:** Conceptualization, Methodology, Project administration, Supervision, Writing – review & editing.

## Declaration of Competing Interest

The authors declare that they have no known competing financial interests or personal relationships that could have appeared to influence the work reported in this paper.

## Data Availability

The data used in this study are available through Lifelines biobank (www.lifelines.nl). Restrictions apply to the availability of these data, which were used under license for this study.
